# On the origin of *POU5F1*

**DOI:** 10.1186/1741-7007-11-56

**Published:** 2013-05-09

**Authors:** Stephen Frankenberg, Marilyn B Renfree

**Affiliations:** 1 Department of Zoology, University of Melbourne, Melbourne, VIC 3010, Australia

## Abstract

**Background:**

Pluripotency is a fundamental property of early mammalian development but it is currently unclear to what extent its cellular mechanisms are conserved in vertebrates or metazoans. POU5F1 and POU2 are the two principle members constituting the class V POU domain family of transcription factors, thought to have a conserved role in the regulation of pluripotency in vertebrates as well as germ cell maintenance and neural patterning. They have undergone a complex pattern of evolution which is poorly understood and controversial.

**Results:**

By analyzing the sequences of *POU5F1*, *POU2* and their flanking genes, we provide strong indirect evidence that *POU5F1* originated at least as early as a common ancestor of gnathostomes but became extinct in a common ancestor of teleost fishes, while both *POU5F1* and *POU2* survived in the sarcopterygian lineage leading to tetrapods. Less divergent forms of *POU5F1* and *POU2* appear to have persisted among cartilaginous fishes.

**Conclusions:**

Our study resolves the controversial evolutionary relationship between teleost *pou2* and tetrapod *POU2* and *POU5F1,* and shows that class V POU transcription factors have existed at least since the common ancestor of gnathostome vertebrates. It provides a framework for elucidating the basis for the lineage-specific extinctions of *POU2* and *POU5F1.*

## Background

Loss of potency during differentiation is fundamental to the development of complex metazoans. Pluripotent embryonic cells are able to give rise ultimately to all tissues of the adult body. In at least some mammals, pluripotency can be “captured” *in vitro* in the form of indefinitely self-renewing embryonic stem (ES) cells. Thus ES cells can serve as a model for the differentiation of their *in vivo* counterparts into ectoderm, mesoderm and endoderm derivatives.

POU5F1 (also called OCT4 or OCT3/4) is a central regulator of pluripotency in mammals. In the mouse, deletion of *Pou5f1* causes loss of pluripotency in the inner cell mass and differentiation to trophoblast, revealing its earliest developmental role [1]. POU5F1 is also a potent reprogramming factor capable of facilitating the derivation of induced pluripotent stem (iPS) cells [2,3]. Conditional knockout of *Pou5f1* in mouse primordial germ cells results in their apoptosis [4], showing that the role of POU5F1 is not exclusively restricted to preventing differentiation.

POU2 is a vertebrate paralog of POU5F1 that has been best characterized in zebrafish. Curiously, some vertebrate lineages, such as salamanders, marsupials and monotremes, have preserved both *POU2* and *POU5F1* in their genomes while in other vertebrates one or the other gene has become extinct [5-7]. Thus squamate reptiles and eutherian mammals have only *POU5F1* while birds and frogs have only *POU2* (called *POUV* in birds). In *Xenopus*, *POU2* is present as three tandem copies - *OCT25*, *OCT60* and *OCT90*.

For reasons that are not fully clear, teleost *pou2* was recently renamed *pou5f1* despite multiple pieces of evidence for a closer affinity to *POU2* orthologs of tetrapods. Onichtchouk [8] argued that since orthologous genes are defined “as originating from a single ancestral gene in the last common ancestor of the compared genomes”, teleost *pou2* is orthologous to mammalian *POU5F1*. However, by the same argument, teleost *pou2* is also orthologous to tetrapod *POU2* orthologs, thus obviating the need for a name change. Teleost *pou2* shares more sequence similarity as well as conserved synteny with tetrapod *POU2*[5,6], but perhaps more importantly, it was not proven whether the duplication event giving rise to each paralog occurred after or before the common ancestor of tetrapods and teleost fishes. If the latter, *POU5F1* must have become extinct in teleosts as it has in some other tetrapod lineages such as birds and frogs.

*POU5F1* and *POU2* share a five-exon genomic structure that is characteristic of the class V POU family. Exons 1 and 5 encode the poorly conserved N- and C-terminal transactivation domains, respectively, while Exons 2 to 4 encode the highly conserved POU-domain, which comprises the POU-specific domain and the POU-homeodomain separated by a short linker region [9-11].

## Results

### Newly identified *POU2* and *POU5F1* orthologs in vertebrates

To gain insight into the origins of the class V POU family of transcription factors in vertebrates, BLAST searches were performed for sequences homologous to mammalian *POU2* and *POU5F1*. Previously unreported orthologs of *POU5F1* were identified from a large number of vertebrate species, including the painted turtle (*Chrysemys picta bellii*), Indian python (*Python molurus*) and coelacanth (*Latimeria chalumnae*). *POU2* orthologs were also identified in many species, including the alligator (*Alligator mississippiensis*), painted turtle, coelacanth and spotted gar (*Lepisosteus oculatus*).

The avian *POU2* ortholog - *POUV* - was identified in genome assemblies of the turkey (*Meleagris gallopavo*), medium ground finch (*Geospiza fortis*) and budgerigar (*Melopsittacus undulatus*), adding to the previously identified orthologs from chicken [12] and zebra finch [5]. Conserved open reading frames orthologous to chicken Exon 1 could not be identified in other avian species. As chicken Exon 1 was previously identified as unlikely to be homologous to Exon 1 from non-avian orthologs [5], all available avian genomes were re-examined. Low stringency BLAST searches identified a single sequence (Ti 224571611) from the chicken whole genome shotgun (WGS) trace archives with homology to the proximal promoter and 5′ part of Exon 1 of non-avian *POU2* orthologs (see below). In addition, a primordial germ cell-derived partial chicken EST (GenBank accession DR410403) included sequence with clear homology to the 3′ part of Exon 1 from non-avian *POU2* orthologs. The apparent absence of both the proximal *POU2* promoter and the “canonical” Exon 1 in other birds is probably due to gaps in their respective genome assemblies, suggesting that features of this region impart recalcitrance to sequencing. We conclude that the previously published cDNA for chicken *POUV* represents a rare or non-canonical chicken-specific transcript (retaining the first intron) that was selectively isolated due to the PCR-based methods used.

**Figure 1:**
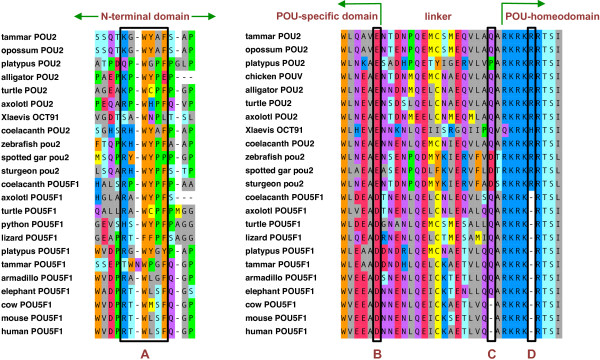
**Sequence signatures among class V POU family proteins.** Within the N-terminal domain, the only motif present in all family members is boxed (**A**). Within the POU domain, position (**B**) is a glutamic acid residue in all POU2 orthologs and an aspartic acid residue in all POU5F1 orthologs. Single-residue deletions at (**C**) and (**D**) are specific to boroeutherian POU5F1 and all POU5F1 orthologs, respectively.

Alignment of a broad selection of *POU2* and *POU5F1* translated sequences (Additional file 1) showed almost no conservation within the N-terminal region between paralogs. However, a short motif with the consensus sequence (K/R)XWYXF was moderately well conserved in both POU2 and POU5F1 (Figure 1), the first time a sequence signature conserved in the N-terminal domain of all family members has been identified. The previously noted N-terminal sequence MAGH and the deletion of a single arginine residue within the POU-homeodomain [5], as well as an aspartic acid instead of glutamic acid at a site within the POU-specific domain identified by Ye and colleagues [13], were among the few fully conserved signatures specifically characterizing POU5F1 orthologs. We also noted that a second single-residue deletion in the linker region separating the POU-specific and POU-homeodomains of POU5F1 is specific to Boreoeutheria, as it was not present in the elephant (Afrotheria), armadillo (Xenarthra) or any other vertebrate (Figure 1), suggesting a modification in function of POU5F1 among some eutherians.

### A multigenic duplication gave rise to *POU2* and *POU5F1*

**Figure 2:**
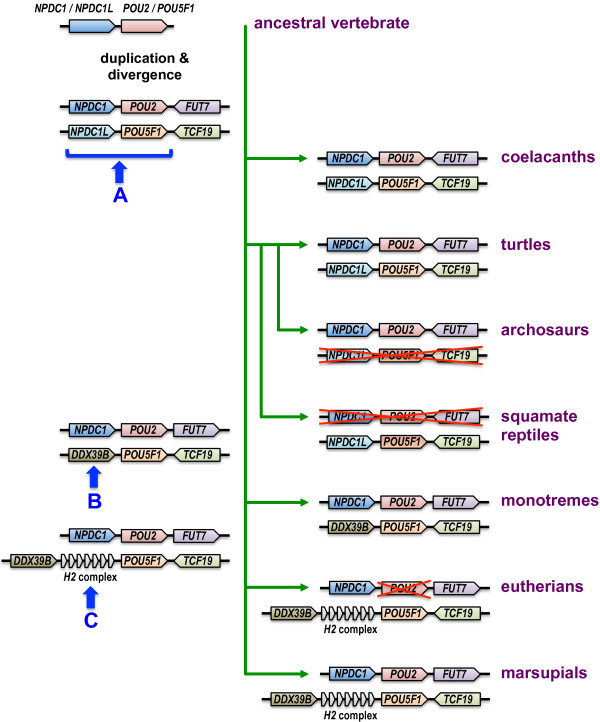
**Synteny at the *POU5F1 *and *POU2 *loci in sarcopterygians.** The synteny of *POU5F1* and *POU2* with other genes in extant species is shown on the right-hand side. Proposed ancestral genomic rearrangements that explain the current synteny are shown on the left-hand side. (**A**) A multigenic duplication in a sarcopterygian ancestor gave rise to *POU5F1* and *NPDC1L*, causing *POU5F1* to flank *TCF19* and *POU2* to flank *FUT7*. (**B**) In a common ancestor of mammals, deletion of *NPDC1L* caused *POU5F1* to flank *DDX39B*. The orientation of *FUT7* also was inverted. (**C**) In a common ancestor of therian mammals, the H2 major histocompatibility complex became inserted between *POU5F1* and *DDX39B*.

**Figure 3:**
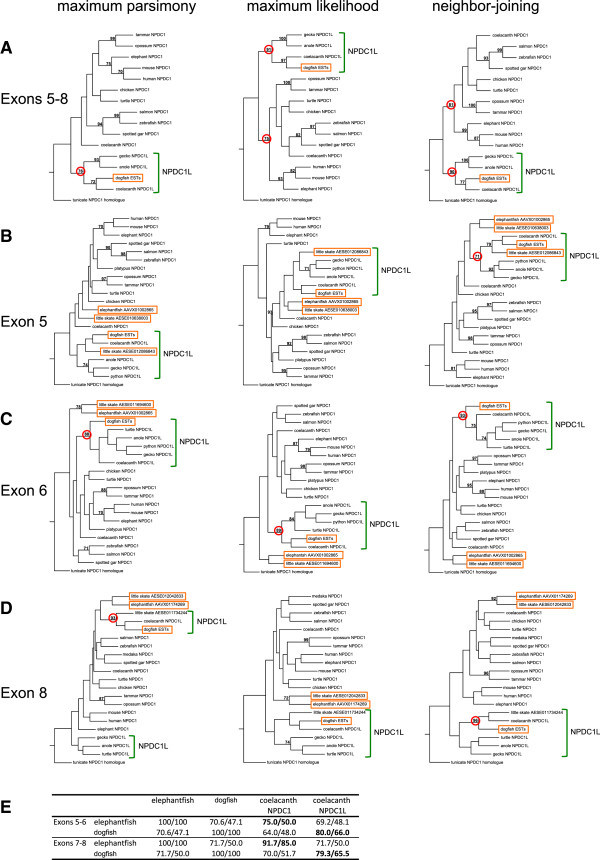
**Phylogenetic analysis of NPDC1/NPDC1L homologs in gnathostomes.** (**A-D**) Phenograms of phylogenetic analyses alignments of translated sequences of *NPDC1*, *NPDC1L* and chondrichthyan sequences analyzed by maximum parsimony, maximum likelihood and neighbor-joining methods, using a tunicate NPDC1/NPDC1L homolog as an outgroup. Alignments are presented in Additional file 2. Only significant bootstrap values (>70%) are shown and those relevant to the text are circled in red. Chondrichthyan sequences are boxed. NPDC1L sequences, including putative chondrichthyan orthologs, are bracketed. (**E**) Percentage of similarity/identity values comparing translated elephantfish and dogfish sequences to each other and to coelacanth NPDC1 and NPDC1L. The percentages highlighted in bold show that the elephantfish sequences are most similar to coelacanth NPDC1 while the dogfish sequence is most similar to coelacanth NPDC1L, indicating respective orthology when combined with the phenogram data.

To gain further insight into the evolution of *POU2* and *POU5F1* and to confirm orthology where possible, we examined their synteny with other genes in available vertebrate genome assemblies (summarized in Figure 2). As previously reported [5,6], *POU2* is flanked by orthologs of *NPDC1* and *FUT7* in all vertebrate genomes for which synteny could be determined. In the coelacanth and turtle genomes, *POU5F1* is flanked by a previously unreported paralog of *NPDC1* - a 9-exon gene expressed in differentiating neuronal cells [14,15] - which we call *NPDC1L* (*NPDC1-like*), indicating that the original duplication event that generated *POU5F1* and *POU2* was of a multigenic region. A search for *NPDC1/NPDC1L* homologs in other vertebrates identified sequences in several squamate reptiles, including python, anole lizard (*Anolis carolinensis*) and Schlegel’s Japanese gecko (*Gekko japonicus*). Phylogenetic analysis showed that these sequences represent *NPDC1L* and not *NPDC1* (Figure 3 and Additional file 2). Thus extinction of *POU2* in squamate reptiles [5] was apparently associated with deletion of a larger, multigenic region that also included *NPDC1*. An additional rearrangement in the mammalian lineage resulted in *DDX39B* lying upstream of *POU5F1* and the possibly simultaneous extinction of *NPDC1L*. In the common ancestor of therian mammals (after their split with monotremes), the *H2* major histocompatibility complex was inserted between *DDX39B* and *POU5F1* (Figure 2).

### The duplication that gave rise to *POU5F1* occurred in a common ancestor of gnathostomes

**Figure 4:**
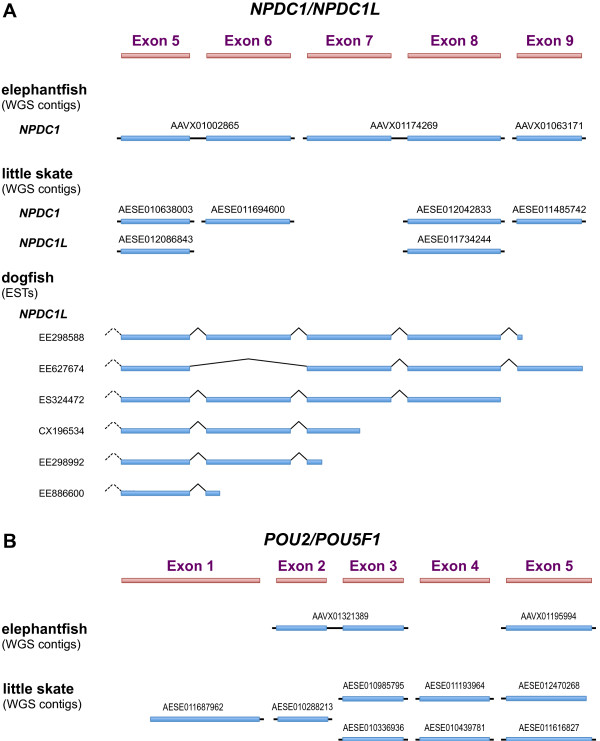
**Summary of genomic and expressed sequences identified in chondrichthyan genomes.** (**A**) Sequences homologous to Exons 5 to 9 of *NPCD1* and *NPDC1L*. (**B**) Sequences homologous to Exons 1 to 5 of *POU2* and *POU5F1*.

The apparent absence of *POU5F1* in all non-sarcopterygian (for example, teleost fish) genomes at first glance suggested that the origin of *POU5F1* by duplication of *POU2* is specific to the sarcopterygian lineage, or at least cannot be proven otherwise. However, the demonstration (above) that the duplication included at least one flanking gene, *NPDC1/NPDC1L*, provided an alternative strategy for determining its timing. We, therefore, searched for homologs of *NPDC1* and *NPDC1L* in cartilaginous fishes (class Chondrichthyes), focusing on Exons 5 to 9 as these are the best conserved. The identified sequences are summarized in Figure 4. Three whole genome shotgun (WGS) contigs were identified from the elephantfish (*Callorhinchus callorynchus*, subclass Holocephali), which included Exons 5 to 6, 7 to 8 and 9, respectively. These presumably form part of a common gene but this was not assumed for the purpose of this analysis. Six WGS contigs were identified from the little skate (*Leucoraja erinacea*, subclass Elasmobranchii), each containing a single exon. These included two homologs of Exon 5, one of Exon 6, two of Exon 8 and one of Exon 9. Thus the little skate genome appears to contain at least two homologs of *NPDC1/NPDC1L*. Lastly, multiple overlapping expressed sequence tags (ESTs) from the spiny dogfish (*Squalus acanthias*, subclass Elasmobranchii) were identified, which together spanned almost the full coding region. These were combined *in silico* to produce a single sequence for analysis.

To maximize statistical power, we first compared the translated dogfish sequence (the only chondrichthyan sequence spanning Exons 5 to 8) with NPDC1 and NPDC1L orthologs of other species, including a tunicate (*Ciona savignyi*) NPDC1/NPDC1L homolog as an outgroup. The dogfish sequence clustered with NPDC1L orthologs with a significant bootstrap value using three different methods for generating consensus phylogenetic trees (maximum parsimony, maximum likelihood and neighbor-joining) (Figure 3A). In a comparison with the sequences from coelacanth, a species with both NPDC1L and NPDC1 (to control for lineage-specific differences in divergence rate), the dogfish sequence was clearly more similar to NPDC1L than to NPDC1, indicating that its clustering with NPDC1L in the consensus trees was not simply due to more rapid divergence from an ancestral NPDC1-like sequence (Figure 3E). This indicated that a gene more similar to *NPDC1L* than to *NPDC1* has existed since at least as early as the common ancestor of Chondrichthyes and Osteichthyes, and that duplication of an *NPDC1*/*NPDC1L* ancestral gene must have occurred before the split between Sarcopterygii and Actinopterygii, since both groups have *NPDC1* orthologs that are more similar to each other than to *NPDC1L*. To examine whether the duplication occurred even earlier in a common ancestor of Chondrichthyes and Osteichthyes, we performed phylogenetic analyses of the other chondrichthyan sequences from elephantfish and little skate (Figure 3B-D). Both of the elephantfish sequences (spanning Exons 5 to 6 and 7 to 8, respectively) clustered with NPCD1 orthologs and were separate from the dogfish sequence and NPDC1L orthologs, regardless of the exon analyzed or the method used. For the little skate, one of the two Exon 5 sequences and one of the two Exon 8 sequences clustered with the dogfish sequence regardless of the analysis method and with significant bootstrap values for three of the six analyses (one for Exon 5 and two for Exon 8), indicating that these sequences are orthologous to the dogfish sequence. The other little skate Exon 5 and Exon 8 sequences, plus the Exon 6 sequence, each clustered with an elephantfish sequence to the exclusion of all other sequences in almost every case (8/9), with only one (non-significant) exception (Exon 5 - maximum parsimony; Figure 3B). Bootstrap values for this clustering were significant in three of the other eight analyses (Exon 6 - maximum parsimony; Exon 8 - maximum likelihood and neighbor-joining). These results strongly suggested that chondrichthyans collectively have both *NPDC1* and *NPDC1L* paralogs and that both are present in the little skate genome. To exclude the possibility that the putative *NPDC1* ortholog (in elephantfish and little skate) is a chondrichthyan-specific paralog of the dogfish sequence, we compared the two elephantfish sequences (Exons 5 to 6 and 7 to 8) to coelacanth NPDC1 and NPDC1L (Figure 3E). Both elephantfish sequences were more similar to coelacanth NPDC1 than to either the dogfish sequence or coelacanth NPDC1L, strongly arguing against a scenario in which the elephantfish sequences are derived from a chondrichthyan-specific duplication of an ancestral *NPDC1*/*NPDC1L* precursor that was more similar to extant *NPDC1L* orthologs than to *NPDC1* orthologs. It may thus be concluded that orthologs of both *NPDC1* and *NPDC1L* are present among cartilaginous fishes and, therefore, that the duplication event giving rise to *POU2* and *POU5F1* must have occurred at least as early as a common ancestor of extant gnathostomes.

### Putative *POU2* and *POU5F1* orthologs are present in chondrichthyans

**Figure 5:**
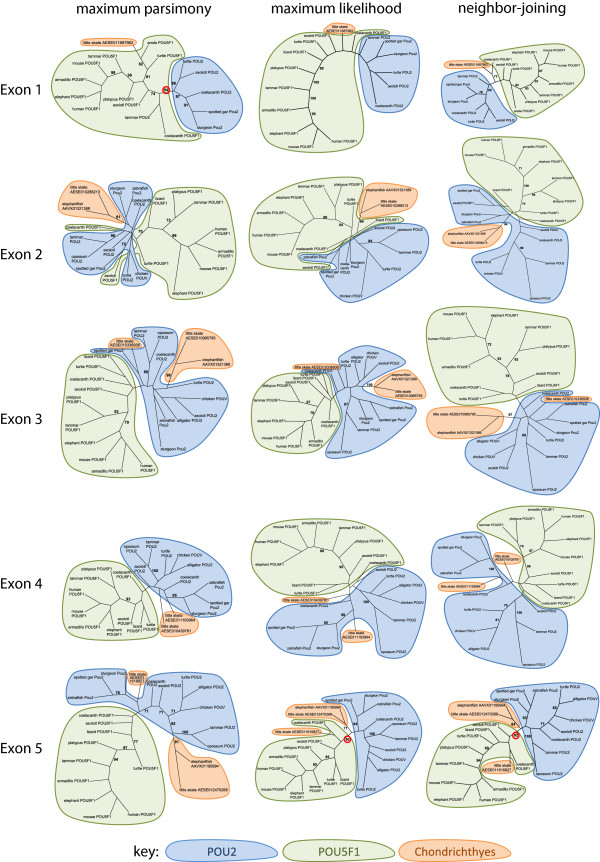
**Phylogenetic analysis of POU2/POU5F1 homologs in gnathostomes.** Translated sequences of individual exons were analyzed by maximum parsimony, maximum likelihood and neighbor-joining methods and displayed as unrooted consensus trees. Alignments for the analyses are presented in Additional file 3. Only significant bootstrap values (>70%) are shown and those relevant to the text are circled in red.

Since the duplication that gave rise to *NPDC1* and *NPDC1L* can be reasonably assumed to have occurred in a common ancestor of cartilaginous fishes and other jawed vertebrates, we searched chondrichthyan databases thoroughly for homologs of *POU2* and *POU5F1*. The identified sequences are summarized in Figure 4. In the elephantfish, we identified a single WGS contig encoding Exons 2 and 3 and a separate contig encoding Exon 5. In the little skate, we identified a partial sequence for Exon 1 and two homologs of each of Exons 3, 4 and 5, all on separate contigs. Thus, while it was unclear which of the identified exons collectively form part of a common gene, at least two genes encoding class V POU domain transcription factors exist in the little skate genome. Although synteny with other genes could not be determined from either of the chondrichthyan genome assemblies, various sequence signatures mostly resembled POU2 rather than POU5F1, including a lack of the single arginine deletion within the POU-specific domain of POU5F1 (Figure 1) [5]. This could be explained by a lineage-specific duplication of *POU2* in the little skate, similar to the tandem *POU2* triplication found in *Xenopus*. However, the presence of a single homolog of both *NPDC1/NPDC1L* and *POU2/POU5F1* in the elephantfish but two homologs of each in the little skate suggested the presence of both *POU2* and *POU5F1* orthologs in the latter species. To test this, we performed phylogenetic analysis of translated sequences for each exon (Figure 5 and Additional file 3). For the elephantfish sequences, Exons 2, 3 and 5 generally clustered with POU2 orthologs and this was highly significant for one analysis of Exon 5 (maximum likelihood; bootstrap value 90%). This suggested that the elephantfish contains a single ortholog of *POU2*. The elephantfish sequences always clustered with one little skate sequence to the exclusion of all others (significant bootstrap value for all three analyses of each exon), indicating orthology. The remaining little skate sequences (Exon1, Exon 3, Exon 4 (×2) and Exon 5) generally clustered non-significantly with either POU2 or POU5F1 orthologs, with two notable exceptions. The little skate Exon 1 sequence clustered with POU5F1 orthologs for all three methods of analysis. For one analysis this was highly significant (maximum parsimony - bootstrap 94%). One of the little skate Exon 5 sequences clustered non-significantly with POU2 orthologs for one analysis method (maximum parsimony) but clustered highly significantly with POU5F1 orthologs for the other two methods (maximum likelihood - 90%; neighbor-joining - 92%).

Combined, the above data suggest that orthologs of both *POU2* and *POU5F1* exist among cartilaginous fishes. Although the identity of every WGS contig cannot be assigned with confidence, evidence suggests that the little skate has both a *POU2* and a *POU5F1* ortholog, while the elephantfish has only a *POU2* ortholog. This is consistent with the presence of both *NPDC1* and *NPDC1L* orthologs in the little skate, but only an *NPDC1L* ortholog in the elephantfish.

## Discussion

Our data show that the duplication that gave rise to *POU5F1* and *POU2* occurred in a common gnathostomal ancestor. This can be deduced by combining two crucial pieces of evidence. First, conserved synteny shows that the duplication was multigenic and also gave rise to the paralogs *NPDC1* and *NPDC1L*. Second, orthologs of both *NPDC1* and *NPDC1L* were identified in cartilaginous fishes. Consistent with this deduction, we identified sequences in cartilaginous fishes that appear to correspond to either *POU2* or *POU5F1*. Orthologs of both *POU2* and *POU5F1* are likely to still be present in the genome of the little skate, although their sequences appear less divergent from each other than they are in higher vertebrates. We also predict that an ortholog of *POU5F1* is present in the spiny dogfish, since this species also retains an ortholog of *NPDC1L*, but *POU5F1* is presumably extinct in the elephantfish lineage.

**Figure 6:**
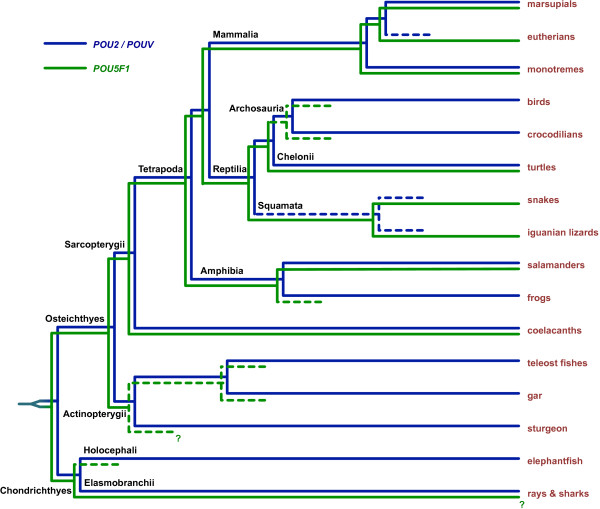
**Model for the evolution of *POU5F1 *and *POU2 *in vertebrates.***POU2* and *POU5F1* arose by duplication of an ancestral gene in a common ancestor of Osteichthyes and Chondrichthyes. One or other gene then became extinct (indicated by dashed lines) in some lineages. The dogfish and little skate are representatives of Elasmobranchii.

A proposed model for the evolution of the *POU2*/*POU5F1* family in vertebrates, based on the present data, is summarized in Figure 6. Turtles, coelacanths and probably at least some elasmobranch fishes all have orthologs of both *POU2* and *POU5F1*, joining with marsupials, monotremes and salamanders [5-7] as the only known lineages that have retained both genes. Extinction of *POU5F1* in birds and crocodilians may have been a single event dating to a common archosaurian ancestor. The absence of *POU2* in both anole and python genomes also suggests a single extinction event in a common ancestor of squamate reptiles.

Contrary to a recent assertion [8], our study provides clear evidence that the *pou2* gene of teleost and other actinopterygian fishes is a *bona fide* ortholog of tetrapod *POU2* and not of *POU5F1*. Its recent renaming to *pou5f1* (RefSeq-ID NM_131112.1) by the zebrafish nomenclature committee is, therefore, misleading. *POU5F1* became extinct possibly in a common ancestor of actinopterygians, or at least of teleost fishes. This finding is important because misleading nomenclature can potentially lead to misleading assumptions regarding evolutionary conservation versus divergence of the respective roles of POU2 and POU5F1.

Orthologs of *POU2* and *POU5F1* from various vertebrates have been tested for their ability to maintain pluripotency in mouse ES cells or to generate mouse or human iPSCs. Non-eutherian *POU5F1* orthologs from axolotl [7] and platypus [6] both have this ability. *POU2* orthologs from opossum, chicken, *Xenopus*, axolotl and medaka are also able to maintain or induce pluripotency [6,7,12,16,17], even in species that have retained both paralogs (axolotl and opossum). Surprisingly, although medaka *pou2* can maintain mESC pluripotency, *pou2* of another teleost fish, zebrafish, cannot [7]. Neverthess, the conservation in function of class V POU family members despite very poor sequence conservation in the transactivation domains can perhaps be expected considering that deletion of either (but not both) of the N- or C-terminal domains did not affect the ability of mouse POU5F1 to maintain ES cell pluripotency [18]. Maintaining pluripotency in ES cells probably serves as a model for only a limited proportion of the roles POU2 and POU5F1 serve *in vivo*. Thus, although there is strong evidence for an ancient role for the common ancestor of *POU2* and *POU5F1* at least in the maintenance of pluripotency, deducing distinct functions and roles between various *POU2* and *POU5F1* orthologs will probably require *in vivo* assays other than ES cell complementation. This would include deducing the function of the conserved (K/R)XWYXF motif in the N-terminal domain.

A general, although not universal pattern, appears to be that *POU2* orthologs are more widely expressed in non-germline and non-pluripotent tissues than are *POU5F1* orthologs. In marsupials, *POU2* transcripts are detectable by RT-PCR in a wide range of adult tissues whereas *POU5F1* expression is restricted to the germ line and early conceptuses [5,19]. Nevertheless, *POU2* is also differentially expressed in early tammar conceptuses [5] and protein immunolocalization suggests that POU2 is a more specific marker than POU5F1 of very early epiblast [20]. Interestingly in the sturgeon, *pou2* transcripts were also detected in many adult tissues [13].

*POU2* orthologs seem to have a more important role than *POU5F1* in early neural development. In the axolotl, *POU2* but not *POU5F1* is expressed specifically in the early neural plate and later in the developing hindbrain [7], in a pattern similar to chicken *POUV*[12], *Xenopus OCT25* and *OCT91*[17] and zebrafish *pou2*[21-25], but not medaka *pou2*[26].

The pattern of germ cell expression is also inconsistent among *POU2* and *POU5F1* orthologs. Marsupial *POU5F1* but not *POU2* expression was detected by *in situ* hybridization in primordial germ-cells and early spermatogonia [5,19], whereas both axolotl paralogs are expressed in germ cells [7]. Germ cell expression has also been reported for chicken *POUV* (*POU2*) and *Xenopus OCT60*[27] and among teleosts for medaka [26] and cod [28] but not for zebrafish. Nevertheless, all *POU5F1* orthologs that have been examined are expressed in germ cells, which may be significant. Two modes of germ cell specification are recognized among vertebrates - predetermined (germ plasm) and inductive (regulative). In the predetermined mode, maternally inherited germ plasm is partitioned during cleavage to a subset of cells, which are then specified to become germ cells. In the inductive mode, there is no germ plasm and germ cells become specified by inducing signals from neighboring cells. The inductive mode is considered ancestral, with the predetermined mode independently derived in birds, frogs and teleost fishes [29]. The predetermined mode was proposed to be correlated with a derived mode of mesoderm induction [30,31] as well as with a more POU5F1-like class V POU transcription factor [32], although this preceded knowledge of the paralogous relationship between *POU2* and *POU5F1* among vertebrates [5,6]. We thus hypothesized that inductive germ cell specification is specifically correlated with the presence of a *POU5F1* ortholog, irrespective of the presence of POU2 [5]. Our present data are still largely consistent with this hypothesis. Evidence suggests that turtles have inductive germ cell specification [33], while retaining *POU5F1* (and *POU2*). To our knowledge, no data exist on the mode of germ cell specification in crocodilians, which would be expected to share a similar mode with birds. Evidence suggests that the sturgeon (a basal actinopterygian) lacks germ plasm and is thus likely to have the inductive mode of germ cell specification [31,34]. The sturgeon genome has not been sequenced, so it is possible that it has a *POU5F1* ortholog in addition to its previously reported *POU2* ortholog [13]. Indeed, Johnson *et al.*[32] do refer to an unpublished “Oct-4” sequence from sturgeon. In the sequenced genome of the spotted gar (a less basal, non-teleost actinopterygian), we found all five exons of a *POU2* ortholog but no exons corresponding to a *POU5F1* ortholog. Thus *POU2* presumably became extinct in a common ancestor of gars and teleost fishes. To our knowledge, the mode of germ cell specification of gars has not been investigated. Early studies of elasmobranch fishes cited by Extavour and Akam [29] drew conflicting conclusions regarding the mode of germ cell specification in elasmobranch fishes and no studies have examined fishes of the subclass Holocephali (for example, elephantfish). Further studies examining the mode of germ cell specification in several of the above lineages will provide powerful data to test the intriguing notion that the acquisition of predetermined germ cell specification permits or even drives the loss of *POU5F1*[32].

## Conclusions

Our study resolves the controversial evolutionary relationship between teleost *pou2* and tetrapod *POU2* and *POU5F1*. It shows that class V POU transcription factors have existed at least since the common ancestor of gnathostome vertebrates and provides a framework for elucidating the basis for the lineage-specific extinctions of *POU2* and *POU5F1*, which is likely to be informative for understanding their roles in development.

## Methods

General sequence analysis was performed using MacVector software, version 12.7.3 (MacVector, Inc.; Cary, North Carolina, USA). Sources of all sequences are detailed in Additional file 4. Sequences were selected to provide a broad range of taxonomic groups. The three *Xenopus* POU2 orthologs (OCT91, OCT60 and OCT25) were not included in analyses since they display considerable sequence divergence, which could be related to redundancy among them. All alignments were performed using the algorithm Muscle (with default parameters) in MacVector on translated sequences. Subsequent manual adjustment was only performed for the full POU2/POU5F1 alignment. Phylogenetic analyses on aligned sequences were performed using PHYLIP version 3.69 [35], using the maximum parsimony (100 replicates, 10 jumbles), maximum likelihood without the assumption of a molecular clock (1,000 replicates, 10 jumbles) and neighbor-joining (1,000 replicates) methods with default parameters.

## Abbreviations

ES cells: Embryonic stem cells; ESTs: Expressed sequence tags; IPSCs: induced pluripotent stemcells; WGS: Whole genome shotgun.

## Competing interests

The authors declare that they have no competing interests.

## Authors’ contributions

SF conceived the study, performed all analyses and drafted the manuscript. SF and MBR contributed to interpretation of the data and the writing of the manuscript. Both authors read and approved the final manuscript.

## Supplementary Material

Additional file 1 **Full alignment of class V POU family translated sequences.** Within the N-terminal domain, the only motif present in all family members is boxed (**A**). Within the POU domain, position (**B**) is a glutamic acid residue in all POU2 orthologs and an aspartic acid residue in all POU5F1 orthologs. Single-residue deletions at (**C**) and (**D**) are specific to boroeutherian POU5F1 and all POU5F1 orthologs, respectively. Click here for file

Additional file 2 **NPDC1 and NPDC1L alignments.** Alignments of NPDC1 and NPDC1L sequences used for phylogenetic analyses. Click here for file

Additional file 3 **POU2 and POU5F1 alignments.** Alignments of class V POU family translated sequences used for phylogenetic analyses. Click here for file

Additional file 4 **Sequence sources.** Sources of all sequences used in this study. Click here for file
