# Supplementary figures and images for: On the origin of POU5F1
Source: BMC Biol. 2013 May 9;11:56. doi: 10.1186/1741-7007-11-56 (PMC3665618; doi:10.1186/1741-7007-11-56)

# Additional file 1

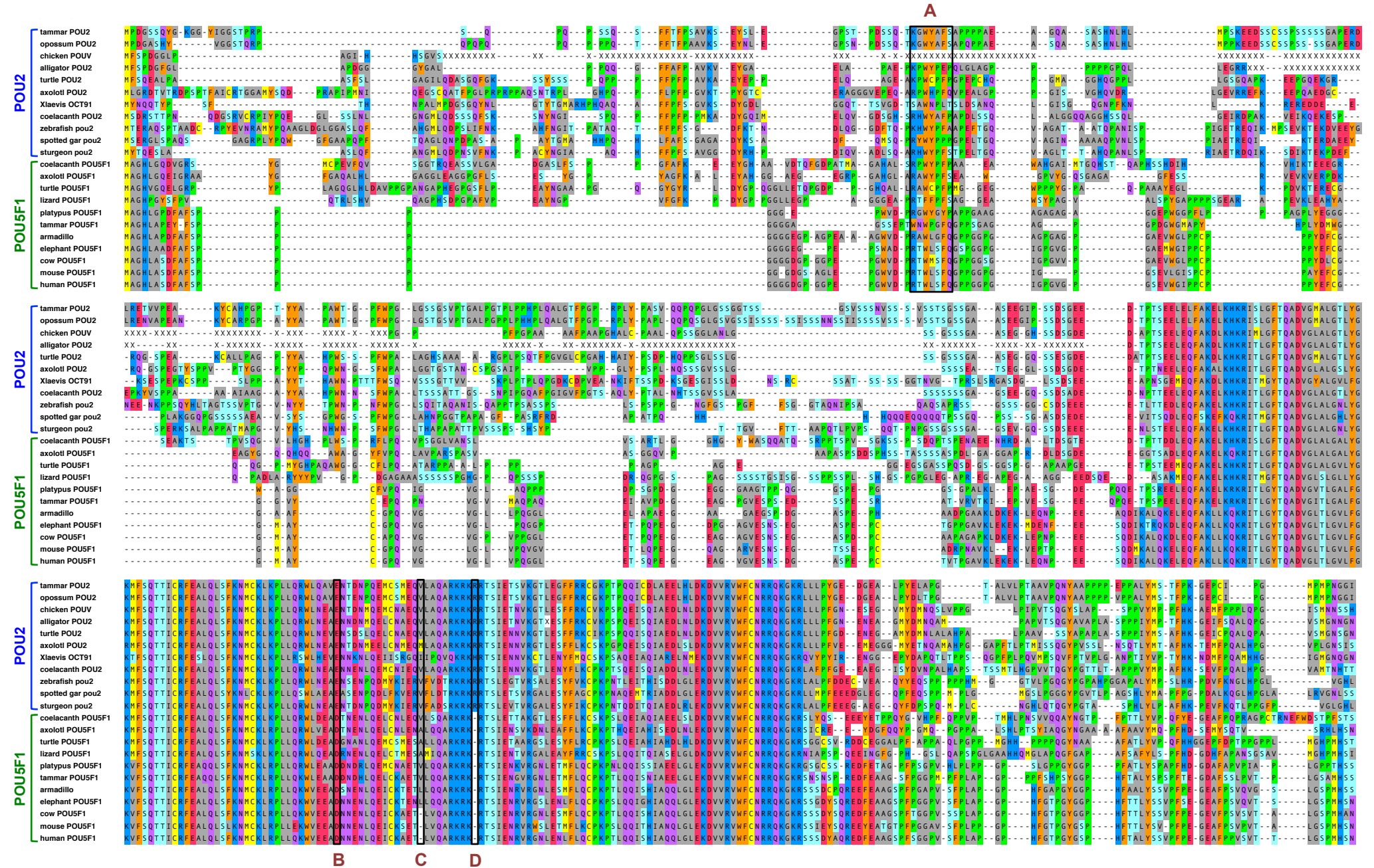

Supplement: Additional file 1 — Full alignment of class V POU family translated sequences. Within the N-terminal domain, the only motif present in all family members is boxed (A). Within the POU domain, position (B) is a glutamic acid residue in all POU2 orthologs and an aspartic acid residue in all POU5F1 orthologs. Single-residue deletions at (C) and (D) are specific to boroeutherian POU5F1 and all POU5F1 orthologs, respectively. [file 1741-7007-11-56-S1.pdf]
