# Supplementary material for: On the origin of POU5F1
Source: BMC Biol. 2013 May 9;11:56. doi: 10.1186/1741-7007-11-56 (PMC3665618; doi:10.1186/1741-7007-11-56)

Additional file 2

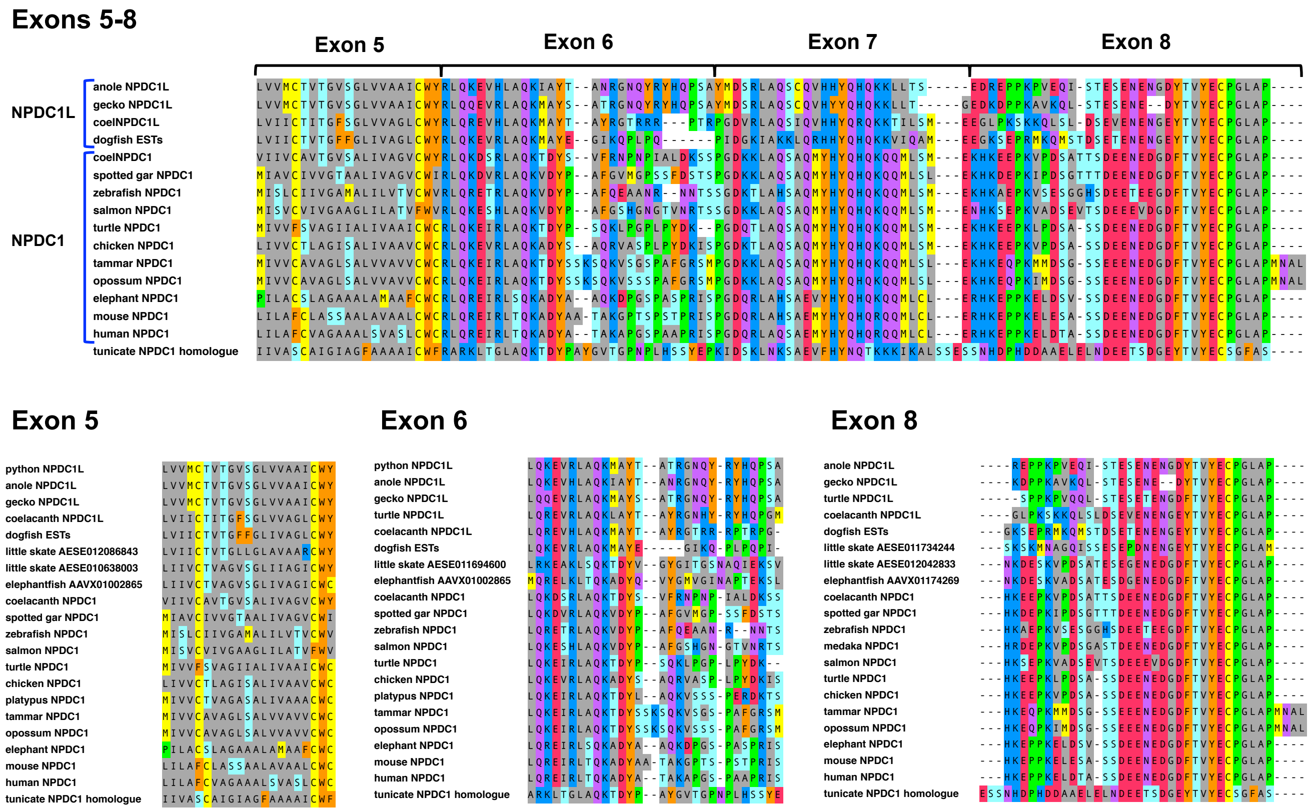

Supplement: Additional file 2 — NPDC1 and NPDC1L alignments. Alignments of NPDC1 and NPDC1L sequences used for phylogenetic analyses. [file 1741-7007-11-56-S2.pdf]
