# Supplementary material for: On the origin of POU5F1
Source: BMC Biol. 2013 May 9;11:56. doi: 10.1186/1741-7007-11-56 (PMC3665618; doi:10.1186/1741-7007-11-56)

Additional file 3

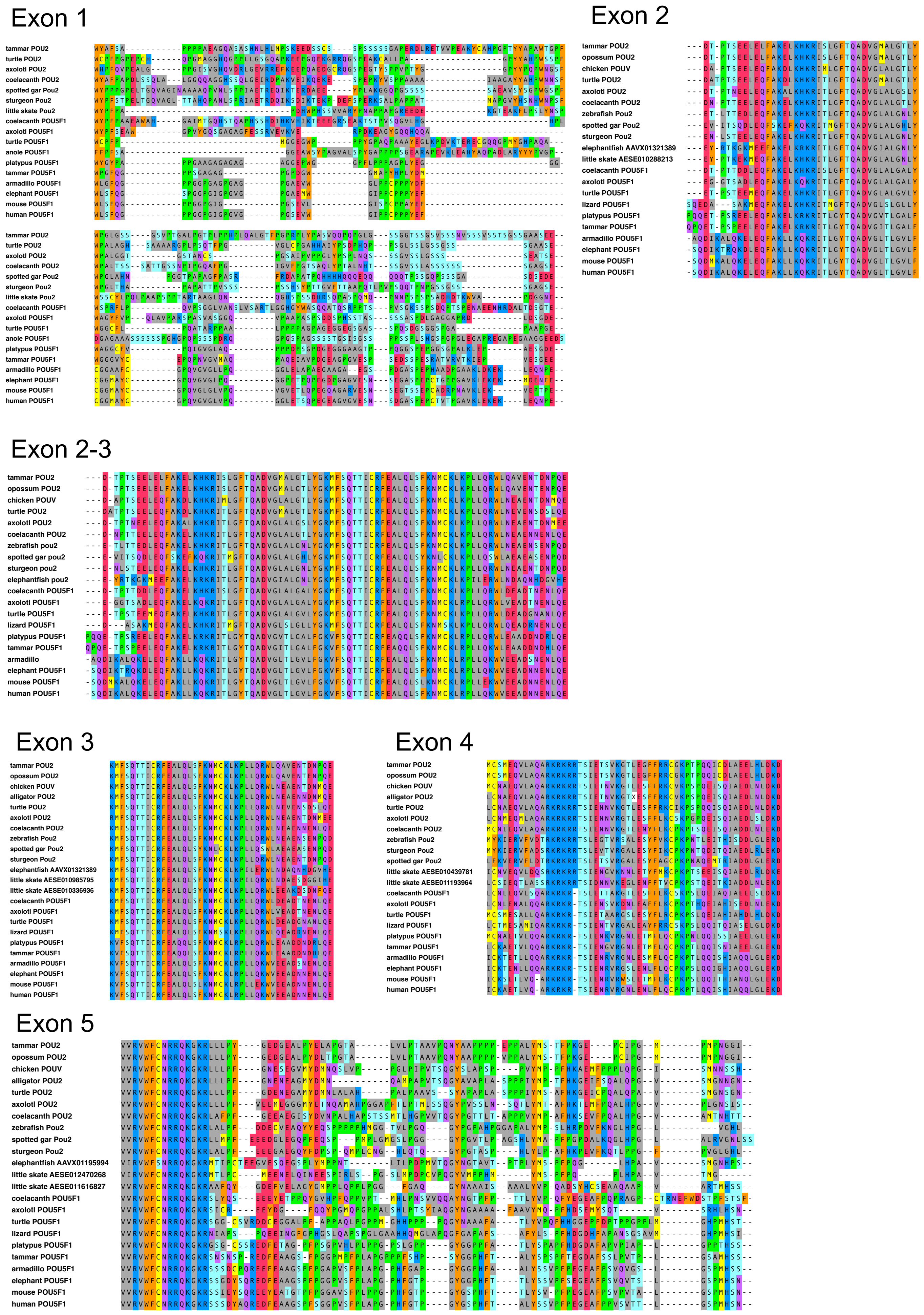

Supplement: Additional file 3 — POU2 and POU5F1 alignments. Alignments of class V POU family translated sequences used for phylogenetic analyses. [file 1741-7007-11-56-S3.pdf]
