# Supplementary material for: On the origin of POU5F1
Source: BMC Biol. 2013 May 9;11:56. doi: 10.1186/1741-7007-11-56 (PMC3665618; doi:10.1186/1741-7007-11-56)
Supplement: Additional file 4 — Sequence sources. Sources of all sequences used in this study. [file 1741-7007-11-56-S4.pdf]

Additional file 4

gene	species	GenBank source
POU2 (& avian *POUV*)	tammar wallaby (*Macropus eugenii*)	FJ998420
	grey short-tailed opossum (*Monodelphis domestica*)	NM_001242727
	chicken (*Gallus gallus*)	partial Exon 1: WGS trace archives Ti:224571611 (62-8, adjusted from chromatogram); DR410403
	alligator (*Alligator mississippiensis*)	partial Exon 1: AKHW01037339 (17977-18192); Exons 2-5: AKHW01037340 (6299-6410, 7612-7742, 11209-11377, 11884-12129)
	painted turtle (*Chrysemys picta bellii*)	AHGY01090879 (23030-23629, 30326-30440, 31140-31270), AHGY01090878 (4146-4310), AHGY01090877 (649-903)
	axolotl (*Ambystoma mexicanum*)	kindly provided by Natalia Tapia
	African clawed frog (*Xenopus laevis*) (*OCT91*)	NM_001087873
	coelacanth (*Latimeria chalumnae*)	JH126767 (739970-739293, 735489-735378, 725094-724964, 718795-718631, 713786-713511)
	zebrafish (*Danio rerio*)	NM_131112
	spotted gar (*Lepisosteus oculatus*)	AHAT01021728 (2445-1807, 595-484, 372-242), AHAT01021727 (2103-1939, 1785-1519)
	sturgeon (*Acipenser sinensis*)	JN099311
POU5F1	human (*Homo sapiens*)	NM_002701
	mouse (*Mus musculus*)	NM_013633
	cow (*Bos taurus*)	NM_174580
	elephant (*Loxodonta africana*)	AAGU03089405 (39093-39485, 42778-42898, 43187-43317, 43645-43806, 43989-44255)
	armadillo (*Dasypus novemcinctus*)	AAGV03091916 (6753-6349-, 4495-4375, 4263-4133, 3996-3835, 3749-3483)
	tammar wallaby (*Macropus eugenii*)	FJ998419
	platypus (*Ornithorhynchus anatinus*)	NM_001242727
	green anole (*Anolis carolinensis*)	AAWZ02031141 (14055-13444, 303-195), AAWZ02031140 (50510-50380, 48892-48731, 47207-46932)
	Indian python (*Python molurus*)	partial Exon 1: AEQU010514509 (1-247); Exons 2-5: AEQU010362508 (1527-1416, 566-436), AEQU010743304 (240-126), AEQU010331488 (1-285)
	painted turtle (*Chrysemys picta bellii*)	AHGY01526669 (432-995), AHGY01303135 (1193-1082, 623-493), AHGY01303134 (178-17), AHGY01303133 (2081-1811)
	axolotl (*Ambystoma mexicanum*)	AY542376
	coelacanth (*Latimeria chalumnae*)	AFYH01090867 (17555-18199), BAHO01146254 (698-809, 2975-3105, 3982-4143, 4560-4850)
Chondrichthyes *POU2/POU5F1* homologues	elephantfish (*Callorhinchus milii*) Exons 2-3	AAVX01321389 (1557-1446, 565-435)
	elephantfish (*Callorhinchus milii*) Exon 5	AAVX01195994 (437-192)
	little skate (*Leucoraja erinacea*) Exon 1 (partial)	AESE011687962 (1-375)
	little skate (*Leucoraja erinacea*) Exon 2	AESE010288213 (446-557)
	little skate (*Leucoraja erinacea*) Exon 3	AESE010985795 (230-100)
	little skate (*Leucoraja erinacea*) Exon 3	AESE010336936 (802-672)
	little skate (*Leucoraja erinacea*) Exon 4	AESE011193964 (297-133)
	little skate (*Leucoraja erinacea*) Exon 4	AESE010439781 (180-16)
	little skate (*Leucoraja erinacea*) Exon 5 (partial)	AESE012470268 (1-220)
	little skate (*Leucoraja erinacea*) Exon 5	AESE011616827 (692-438)
NPDC1 (Exons 5-9)	coelacanth (*Latimeria chalumnae*)	AFYH01056513 (9699-9633, 8270-8186, 6195-6116, 4750-4651), AFYH01056512 (9547-9470)
	spotted gar (*Lepisosteus oculatus*)	AHAT01021729 (5684-5618, 4889-4805, 4688-4609, 4472-4373, 3219-3148)
	zebrafish (*Danio rerio*)	NM_001128783
	medaka (*Oryzias latipes*)	AM301011
	salmon (*Salmo salar*)	NM_001173856
	painted turtle (*Chrysemys picta bellii*) (Exons 5-9)	AHGY01090880 (17417-17482, 18922-19000, 19260-19339, 19642-19738), AHGY01090879 (503-580)
	chicken (*Gallus gallus*)	XM_001233958
	platypus (*Ornithorhynchus anatinus*) (Exons 5-7)	AAPN01166064 (2178-2112, 1875-1791, 687-608)
	tammar wallaby (*Macropus eugenii*)	FY568867
	grey short-tailed opossum (*Monodelphis domestica*)	XM_001374335
	elephant (*Loxodonta africana*)	AAGU03092129 (4108-4174, 4253-4337, 4418-4497, 4742-4838)
	mouse (*Mus musculus*)	NM_008721
	human (*Homo sapiens*)	NM_015392
	elephantfish (*Callorhinchus milii*) Exons 5-6	AAVX01002865 (2746-2812, 3205-3293)
	elephantfish (*Callorhinchus milii*) Exons 7-8	AAVX01174269 (965-886, 570-471)
	elephantfish (*Callorhinchus milii*) Exons 9	AAVX01063171 (561-638)
	little skate (*Leucoraja erinacea*) Exon 5	AESE010638003 (205-271)
	little skate (*Leucoraja erinacea*) Exon 6	AESE011694600 (587-500)
	little skate (*Leucoraja erinacea*) Exon 8	AESE012042833 (107-206)
	little skate (*Leucoraja erinacea*) Exon 9	AESE011485742 (1535-1464)
NPDC1L (Exons 5-9)	Japanese gecko (*Gekko japonicus*)	EB172012 (1-523)
	Indian python (*Python morulus*) (Exons 5-7)	AEQU010264385 (1694-1628, 1356-1272, 828-752)
	green anole (*Anolis carolinensis*)	WGS trace archives Ti:1411938372 (339-405), AAWZ02031143 (3431-3347, 1639-1563), AAWZ02037787 (11785-11692, 7770-7705)
	painted turtle (*Chrysemys picta bellii*) (Exons 6-8)	AHGY01303137 (1423-1339, 1069-993, 262-172)
	coelacanth (*Latimeria chalumnae*)	AFYH01090867 (3719-3785, 5136-5211, 6685-6764, 8172-8268, 9231-9308)
	dogfish (*Squalus acanthias*)	EE298588, EE627674, ES324472, CX196534, EE298992, EE886600
	little skate (*Leucoraja erinacea*) Exon 5	AESE012086843 (111-45)
	little skate (*Leucoraja erinacea*) Exon 8	AESE011734244 (193-94)
tunicate *NPDC1/NPDC1L* homologue	tunicate (*Ciona savignyi*) (Exons 5-9)	AACT01015729 (12830-12896, 14325-14415, 15427-15506, 15995-16103, 16335-16481)
